# A Framework for Context Sensitive Risk-Based Access Control in Medical Information Systems

**DOI:** 10.1155/2015/265132

**Published:** 2015-05-04

**Authors:** Donghee Choi, Dohoon Kim, Seog Park

**Affiliations:** ^1^Computer Science and Engineering, Sogang University, Seoul 121-742, Republic of Korea; ^2^Agency for Defense Development, Daejeon 305-600, Republic of Korea

## Abstract

Since the access control environment has changed and the threat of insider information leakage has come to the fore, studies on risk-based access control models that decide access permissions dynamically have been conducted vigorously. Medical information systems should protect sensitive data such as medical information from insider threat and enable dynamic access control depending on the context such as life-threatening emergencies. In this paper, we suggest an approach and framework for context sensitive risk-based access control suitable for medical information systems. This approach categorizes context information, estimating and applying risk through context- and treatment-based permission profiling and specifications by expanding the eXtensible Access Control Markup Language (XACML) to apply risk. The proposed framework supports quick responses to medical situations and prevents unnecessary insider data access through dynamic access authorization decisions in accordance with the severity of the context and treatment.

## 1. Introduction

Rapid changes and developments to computer information systems have brought about various changes in medical environments. Radiofrequency identification (RFID), ubiquitous sensor networks (USN), and wired/wireless networks have since become integrated into medical environments, improving accessibility and convenience. Moreover, a conversion to the so-called U-Health system is under way [1].

Rapid changes to information technology have affected information access environments in various ways. The collection and analysis of various and massive information are now possible, and information access environments, subjects, objects, and purposes have diversified. Role-based access control (RBAC) [2, 3] is widely adopted for access control in information systems. However, RBAC predefines access policies, and access control decisions are exclusively made on the basis of these predefined polices [4]. Consequently, it is impossible to apply dynamic access control to various access environments, such as wireless connections and emergencies.

To solve this problem, risk-based access control has been introduced [5]. Risk-based access control evaluates risk by considering the access request environment and situation [6], along with the security policies, and decides the access permissions according to a threshold, below which there is an acceptable level of risk [7, 8]. This manner for deciding access permissions makes dynamic access control possible by reflecting the nature of the situation and by preventing unnecessary information access and leakage caused by the misuse and abuse of data by insiders [9].

Medical Information systems (MISs) must consider several features with regard to access control. First, there are various emergency situations in medical environments, and it is crucial to respond to such situations promptly [10]. Therefore, the situation must be taken into consideration when deciding access permissions, and access decisions should change, depending on the severity of the situation [11]. Second, MISs store sensitive data, such as personal information and medical records, and information leakages in this field are likely to result in egregious violations of patient confidentiality. Thus, it is important to prevent information leakages caused by the misuse and abuse of authorized users by controlling their access. Finally, MISs deal with various symptoms and treatments, and the required data differs depending on these symptoms and treatments. However, it is not easy for a security policy administrator who does not have medical expertise to predefine security policies on this basis [12]. Defining a security policy for an MIS requires collecting and analyzing the information access history and profiling the access permissions based on actual symptoms and treatments, rather than merely the administrator's specifications of the security policy.

For access control that reflects the features of an MIS, this paper proposes a context sensitive access control framework that evaluates and applies risk through treatment-based permission profiling. The contributions of this paper are as follows:

- Proposing a framework for context sensitive risk-based access control over MISs.
- Categorizing the contexts that occur in medical environments by severity and enabling dynamic access control according to severity by setting different risk thresholds.
- Establishing a treatment dependent permission profile for accessing data and proposing a method for estimating risk based on correlations with this permission profile.
- Identifying the risk-applicable components in RBAC and describing the interactions between contexts and risk-applicable components.
- Defining the Policy Risk Point, an additional component in the XACML [13] for applying risk [14], and providing specifications for expanding the XACML.

The paper is organized as follows.  Section 2 explains the related studies.  Section 3 concerns the framework for context sensitive risk-based access control.  Section 4 describes the XACML 3.0-based formal specifications for the proposed framework, and Section 5 presents our conclusions.

## 2. Related Work

A number of studies have been conducted on access control systems that apply a risk awareness approach for dynamic access control. According to [12], access to unnecessary data for corresponding job functions is assigned a higher risk score, whereas access to necessary data has a lower risk score. This model defines the correlation between the medical record and the access purpose and decides access permissions in terms of providing access to less risky data by taking into consideration this correlation.

Furthermore, [15] proposed a risk-aware RBAC session that applies risk to role-based access control. This approach makes dynamic user authorization decisions in a session based on the risk inherent in the current situation. The main idea is to set the maximum limit for user access to a certain session as the risk threshold. Risk thresholds are classified as static, dynamic, or adaptive, depending on estimations of the time and risk involved. This proposal included a framework that activates and deactivates role assignment functions according to these risk thresholds.

In [16], a model is proposed for estimating risk and inducing fuzziness using a Bell-Lapadula model for access control. Similarly, [17] proposed attribute-based access control that takes risk into consideration. This model includes the concept of risk in user control and considers the risk originating from users, objects, calculations, and connections, and the trust originating from attribute certificates.

There have been other proposals for access control models that take into account situations in need of rapid response in medical environments. For instance, [18] proposed an access control model for prompt responses to emergency situations in medical environments. This model makes it possible to cope with rapidly changing situations by analyzing these situations step by step in accordance with priorities and by establishing the appropriate policies and permissions for particular situations.

According to the risk estimation in traditional risk-based access control models, a user's risk score increases when data is more sensitive and with a higher number of data accesses. Thus, users whose jobs require frequent access to sensitive data have higher accumulated risks, and access to certain data is restricted to users who require such access for their work. However, access to sensitive data occurs frequently in medical environments, depending on the type of disease and treatment. Therefore, a traditional risk estimation approach is unsuitable in a MIS.

In this paper, we apply risk-based access control for dynamic access control and propose a framework for risk-based access control that is suitable for medical environments by utilizing data access information based on the severity of the patient's condition and the type of treatment that is prescribed.

## 3. Framework for Context Sensitive Risk-Based Access Control

This section provides an overview for the proposed framework. It discusses the classification of contexts, the treatment-based permission profiles, risk assessment, the inclusion of risk into RBAC, and the framework for context sensitive risk-based access control in an MIS.

### 3.1. Overview of the Proposed Framework

When accessing information systems in medical environments, the patient data that is required differs depending on the severity of the patient's condition and treatment. The access control system predefines access policies and decides permissions and rejections according to the access permission defined when requested by the user. However, this type of access control does not reflect the characteristics of medical environments, and, in turn, it does not reflect the severity of the patient's condition nor the type of treatment. Rather, it simply decides access permissions and rejections according to the predefined access policy.

This paper proposes a framework for context sensitive risk-based access control, providing dynamic access control in MISs in accordance with the severity of the patient's condition and treatment. The proposed framework evaluates the risk of the user's request and grants permission only when the request falls below an acceptable level of risk. Evaluating the risk in requested data requires a consideration of the various treatments and medical information. The risk is low when the requested data is necessary for the corresponding treatment, and the risk is high when the requested data is unnecessary. A treatment-based permission profile is established as a permission set that contains the accumulated and analyzed data access history for all doctors, sorted according to the type of treatment. When data access is requested, the risk score is determined according to the correlation between the requested data and the permission profile.

Contexts are classified as critical, serious, and stable, depending on the severity of patient's condition, reflecting the context for access control. The classified severity levels are used to decide the risk threshold—that is, the acceptable level of risk for access control. In severe cases, the risk threshold is correspondingly high, permitting access to high-risk data. For example, if the patient's severity level is “critical,” access is granted to the requested data regardless of the risk. Doctors will receive the information immediately, in hopes of preventing dangerous situations that can affect the patient's life. Thus, higher priority is assigned to the patients' safety than to the protection of data when the patient's condition is critical. When the level is set to “serious,” the risk threshold is higher than it is when the patient's condition is “stable.” Therefore it is possible to determine access permissions dynamically according to the severity of the patient's condition.

In this paper, an additional component is defined and specifications are provided for implementing an XACML-based approach. In addition, a Policy Risk Point (PRP) is defined to evaluate risk in medical environments. The PRP estimates risk by obtaining Policy Information Point (PIP) information from the context handler, subsequently returning the results to the context handler. The context handler then delivers these results to the Policy Decision Point (PDP) to use it for access control.

The PRP is composed of the profiler engine, the permission profile, and the risk calculator. The profiler engine creates a treatment-based permission profile with the access history logs. The risk calculator evaluates risk by analyzing the correlation between the requested data and the profile, according to patient's condition and treatment, and delivers the results to the context handler. The context handler delivers the related information to the PDP, which decides access permissions by taking into account the context level and the risk when evaluating access requests. The details for the proposed framework are as follows.

### 3.2. Classification of Contexts

**Figure 1:**
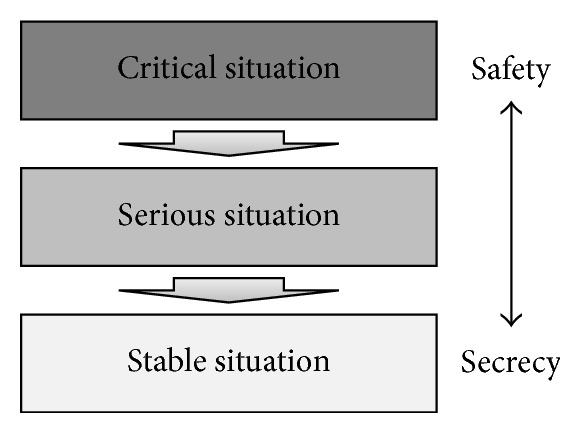
Severity levels.

In medical environments, life-threatening emergencies are common, and these situations require prompt and accurate responses. To cope with sudden changes in a patient's condition, access permissions must be decided quickly and accurately. The proposed framework actively reflects situational contexts in medical environments by classifying medical contexts according to the degree of severity and with dynamic access control according to the context. Contexts are classified by reference to [17].  Figure 1 shows the context classification according to the severity.

Contexts are classified into critical, serious, and stable, and different risk thresholds are applied for different access permissions by severity. Here, a “threshold” refers to the acceptable level of risk. In a high severity context, where priority is placed on the safety of patients rather than data leakage, the threshold is high. In a low severity or “stable” context, the threshold is low, emphasizing the protection of sensitive data. 

*(i) “Critical” Situations.* The context is “critical” when the patient's life is in danger, such as when the patient's heart rate drops to zero. This context is assigned the highest degree of severity. Consequently, the patients' safety is paramount, and access is granted for requested data without any assessment of the risk. Doctors receive information immediately, and it is hoped that, with this information, they can better address the life-threatening situation. Importantly, the user's accessed data is logged for auditing, and after the critical situation ends, this data is studied to determine whether it was accessed legally. If it was not, the user must perform the obligations. Thus, when the situation is critical, the patient's safety always trumps concerns for protecting data, at least momentarily. 

*(ii) “Serious” Situations.* In this situation, the patient's condition is urgent, such as when the patient's heart rate drops by more than 30 beats per minute for more than two minutes. In this situation, the severity level is higher than “stable,” and doctors can access data that is otherwise prohibited. For example, in “serious” situations, doctors can access patient data from other departments. Thus, in this context, access is granted to riskier data in order to expedite the patient's treatment. 

*(iii) “Stable” Situations.* When the situation is stable, the patient's condition is normal. Users are permitted access only to authorized data. Data protection is emphasized in order to prevent information leaks, because the patient is not facing a life-threatening situation.

A tradeoff between safety and security therefore exists depending on the severity of an illness. Because different risk thresholds are assigned, depending on the degree of severity, dynamic and context-aware risk application is possible. Contexts are classified as such and applied to the configuration of the permission profile created based on the data access history. These profiles are used as a reference to confirm the severity when users request access to data.

### 3.3. Treatment-Based Permission Profile

Doctors use different treatments depending on a patient's symptoms and condition, and access to certain patient information should be determined based on the type of treatment. In typical MISs, doctors are granted high-level access to most of the information regarding their patients. However, this comes at the cost of a higher probability of information leakage. Medical information leaks have serious consequences.

To prevent these leaks, doctors' access to patient information can be limited to the data they require in the current context, given the symptoms and treatment. This follows the so-called principle of least privilege. For example, for pulmonary edema, the applicable treatments are the administration of oxygen, antihypertensive, nitroglycerine, and so forth. Because the information required for each treatment is available, the doctor shall be granted access only to the data necessary for the corresponding treatment.

It is difficult to predefine a security policy that describes the treatments for certain symptoms in certain situations, with the information required for this treatment [18]. Because there are countless symptoms, situations, and treatments, it is troublesome for security experts to predefine the corresponding information. To solve this problem, the doctors in a medical environment can gather to analyze the access logs of the information associated with each type of treatment for their patients' respective symptoms and contexts, and a treatment-based permission profile can be established based on these logs.

**Figure 2:**
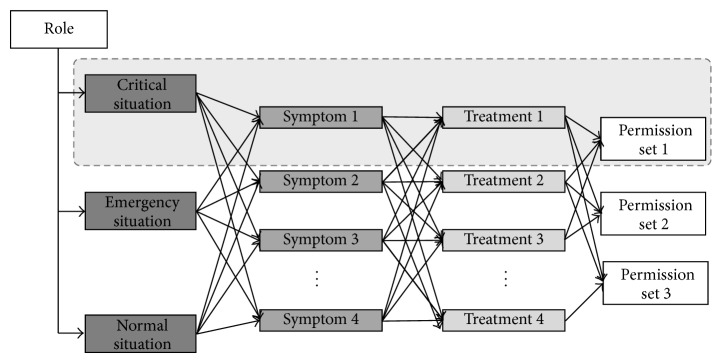
Treatment-based permission profile.

Figure 2 shows the correlations between situations, symptoms, and treatments in a permission profile. Permission profiling is used to classify contexts into critical, serious, and stable, according to severity, and to configure a group of data accessed according to symptoms and treatments in each context as a permission set. Because permission profiling is configured based on the data access history after the learning period, only the access permissions actually required to perform the job functions are identified under the need-to-know principle, and thus permission profiling reduces unnecessary data access and prevents insider data misuse and abuse and information leakages.

### 3.4. Risk Assessment

In general risk-based access control, risk is evaluated according to the frequency of access to sensitive data. However, MISs require continual access to highly sensitive patient information for the purpose of treatment.

Were the general risk evaluation methods applied to MISs without modification, the data access for doctors who request data frequently would be rejected, because their risk scores accumulate, even when they attempt to access data legally. To resolve this problem, a risk evaluation method that reflects the characteristics of medical environments is required. Because doctors request access to different data, depending on the severity of the patient's condition, symptoms, and treatments, a treatment-based permission profile is established, and risk is estimated by reference to this permission profile.

When doctors request access to information based on a patient's symptoms and situation, the risk is the degree by which the requested data disagrees with the profile compared to the permission set from the treatment-based permission profile. Risk thresholds differ depending on the severity of the patient's condition. If the level of risk falls below the threshold, then access is granted. This type of risk evaluation is more realistic and suitable to medical environments than risk evaluations based on data sensitivity and access frequency, which are used in traditional risk-based access control.

### 3.5. Role-Based Access Control (RBAC) with Risk

The proposed framework, context sensitive risk-based access control (CSRAC), is based on the RBAC model. RBAC is composed of the user, role, session, user-role assignment (URA), permission-role assignment (PRA), role hierarchy, and constraints. Users activate the roles given to them (i.e., from the URA) through the session. Permissions are assigned differently according to the roles activated by users. Users are assigned permissions (i.e., from the PRA) contained in the activated roles.

**Figure 3:**
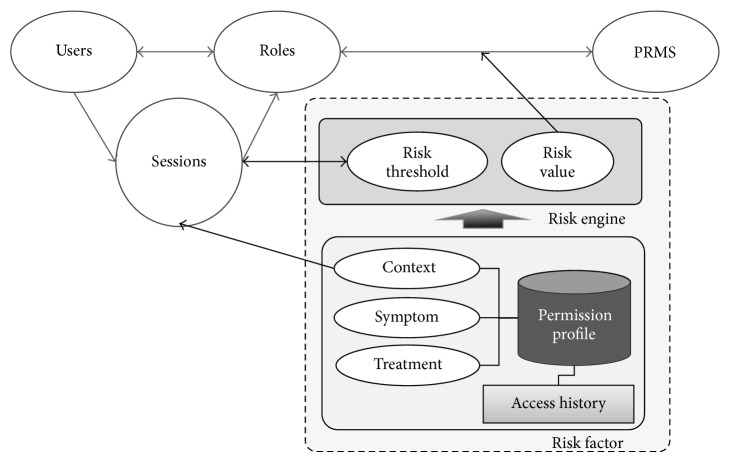
Context sensitive risk-based access control.

Figure 3 shows the RBAC risk-application components for the proposed CSRAC. Risk estimation with CSRAC is determined according to the severity of contexts and its correlation with the permission profile. The severity levels are reflected by applying the threshold to the session components. The risk is applied by PRA components. As explained in Section 3.1, the session components classify the severity levels for the contexts and adjust the corresponding threshold. The risk threshold is higher as the context is more severe, and data access is granted, assigning higher priority to treatment than data protection in life-threatening situations. If the severity level is low—for example, in “stable” contexts—the risk threshold is likewise low, blocking access to high-risk data. In this way, the proposed framework enables dynamic access control according to the severity of the context and whether necessary data has been requested.

### 3.6. Framework for Context Sensitive Risk-Based Access Control in an MIS

To apply the context sensitive risk-based control in MISs, additional information is required concerning the contexts, symptoms, and treatments. Accumulations and analyses of the access history constitute part of this additional information, along with the configuration of the permission profile, confirmation of the context, symptoms, and treatment information for the corresponding session, and a comparison with the permission profile. Because the permission profile is established based on the access history, a request for information that is not included in the permission profile implies that such information is unlikely to be essential for understanding the patient's condition, symptoms, and treatments.

**Figure 4:**
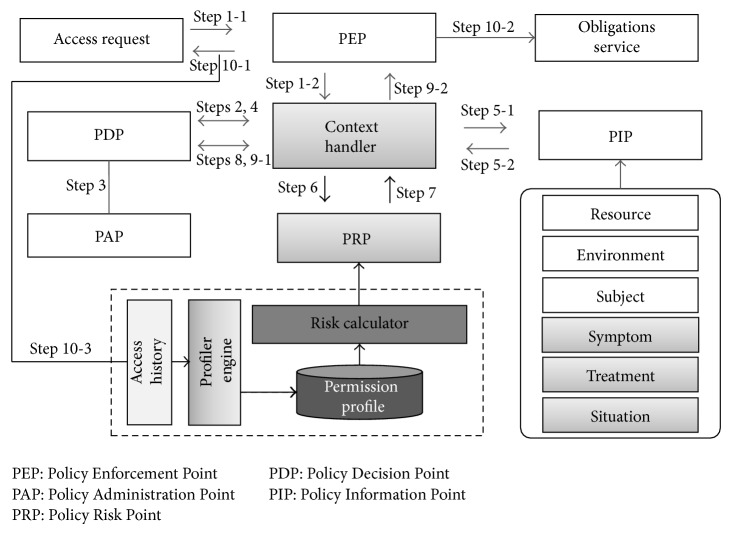
Framework for CSRAC.

Therefore, the risk estimation for MISs is decided according to the degree of correlation between the requested data and the permission profile. If the requested data is highly correlated with the profile data, the risk decreases. If the correlation is low, the risk increases. In this way, risk-based access control is suitable for MISs when estimating the risk in consideration of context, symptoms, and treatments.  Figure 4 shows the proposed CSRAC framework. This framework expands the components based in the XACML for risk application.

The components added to the XACML for the proposed framework are the expansion of PIP information and the PRP module.

- Policy Risk Point (PRP): the PRP is a module complementing the PAP and the context handler, and it is taken into account when deciding access permissions. The profiler engine creates the permission profile database with the context, symptom, and treatment data in the access history. The risk calculator estimates the risk using the PIP information and delivers the result to the PDP through the permission profile database. The PDP uses it when deciding access permissions.
- To decide access permissions, the PIP obtains the information regarding the access request and delivers the resource information to the context handler. The symptom and treatment information essential for risk estimation is also collected and delivered to the PIP. The PIP then delivers the corresponding information to the PRP to estimate the risk.
- Once the acceptability of the access request is decided, the PEP delivers the decision regarding access permission to the user. The access history is delivered to the PRP and used for permission profiling through the profiler engine.

The information flow for the proposed framework that expands the XACML by applying risk for MISs is as follows.

**Step 1 .**

The PEP receives each access request and delivers it to the context handler.

**Step 2 .**

The context handler converts the received request into the XACML request context and delivers it to the PDP.

**Step 3 .**

The PDP evaluates the request through the related XACML policy queries stored in the PAP.

**Step 4 .**

If the corresponding policy references additional attributes that are not included in the requested context, the PDP requests from the context handler the corresponding attributes and risk value for the request. If the severity level is “critical,” then skip to Step 8.

**Step 5 .**

The context handler requests from the PIP the attributes required for a risk estimation of the request, and the PIP provides the corresponding information.

**Step 6 .**

The context handler delivers the request and the attributes received from the PIP to the PRP and requests the risk value.

**Step 7 .**

The PRP calculates the risk based on the attributes received from the context handler and the severity of the context and delivers the risk value to the context handler.

**Step 8 .**

The context handler delivers the requested attributes and the risk value to the PDP.

**Step 9 .**

The PDP evaluates the policy and provides a response to the PEP.

**Step 10 .**

The PEP performs authorization decisions and executes obligations.

## 4. Formal Specifications

OASIS released the eXtensible Access Control Markup Language (XACML), an XML-based standard for specifying and evaluating access control policies. The XACML supports the policy exchange format standard and fine-grained authorization policies independently from implementation. The XACML's RBAC profile (RB-XACML) was proposed for implementing the XACML for role-based access control, and it is designed for the core and hierarchy components in RBAC. The formal specifications for the proposed framework are based on the XACML's RBAC profile.

The specifications for user access requests and security policies are as follows. The requested context level and treatment are defined as additional attributes in Box 1. This way, the risk and the threshold are evaluated in the PRP. The policy stipulates that access will be granted only when the risk value is lower than the threshold, in order to consider risk when evaluating PDP access requests in Box 2.

## 5. Conclusion

The access control model used in traditional MISs decides whether access is granted according to predefined permissions. It does not consider the patient's condition or treatments that occur in medical environments. The concern is that, with this model, access is often granted to unnecessary information, and the need-to-know principle is not observed.

In this paper, we proposed a framework for context sensitive risk-based access control, providing dynamic access control such that only the information required for treating the patient's current condition will be provided when accessing the patient's information in an MIS. To consider the patient's condition in a medical environment, the proposed framework classifies contexts into critical, serious, and stable and supports dynamic access control according to the severity of the context. In addition, we established a treatment-based permission profile by accumulating and analyzing the data access history. If data that is not included in the profile is requested, the request is considered to pose a high risk. In this way, we prevent access to data that is unnecessary for specific treatments. To implement the XACML-based approach, additional components were defined, and these were represented as specifications. By estimating the risk with a treatment-based permission profile when users request data and by deciding the acceptable level of risk based on the patient's condition, access control is dynamic and tied to the patient's condition and treatment. This makes it possible to provide fine-grained access control to sensitive medical information and to prevent insider data misuse and abuse by restricting even authorized users from accessing information unrelated to treatment—that is, by permitting access under the need-to-know principle. In the future, we plan to investigate specific methods for treatment-based permission profiling based on an analysis of the access history information. Moreover, we shall subject our proposal to a performance evaluation to establish its feasibility in calculating risk according to a severity threshold.

## Figures and Tables

**Box 1:**
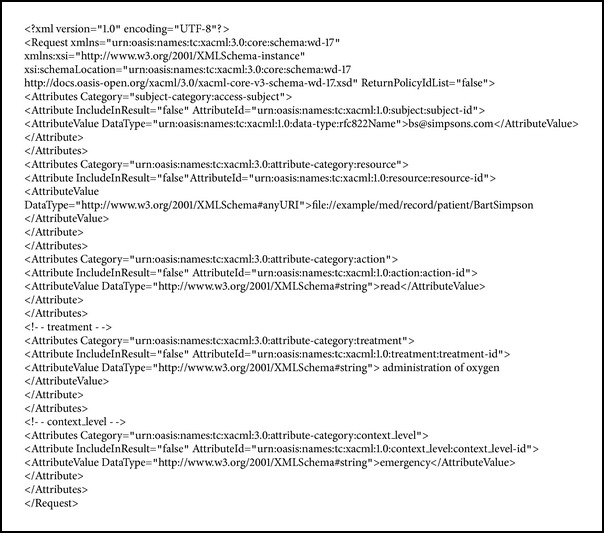
**Box 1: **Specifications for user requests.

**Box 2:**
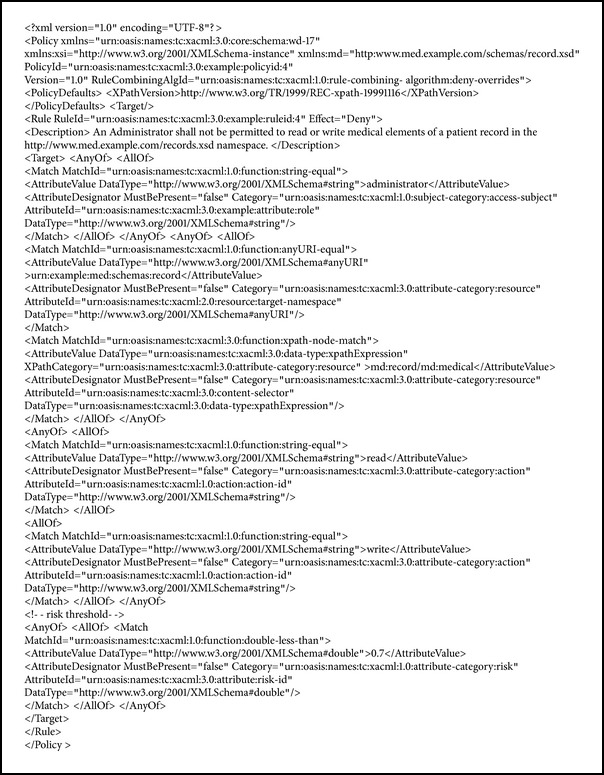
**Box 2: **Specifications for the security policy.
